# Trends in use of sodium-glucose co-transporter 2 inhibitors (SGLT2i) and glucagon-like peptide-1 receptor agonists (GLP-1RA) in Australia in the era of increased evidence of their cardiovascular benefits (2014–2022)

**DOI:** 10.1007/s00228-023-03539-8

**Published:** 2023-07-14

**Authors:** Jialing Lin, Sallie-Anne Pearson, Jerry R. Greenfield, Kyeong Hye Park, Alys Havard, David Brieger, Richard O. Day, Michael O. Falster, Juliana de Oliveira Costa

**Affiliations:** 1 Medicines Intelligence Research Program, School of Population Health, Faculty of Medicine and Health, University of New South Wales, Sydney, Australia; 2 Department of Diabetes and Endocrinology, St Vincent’s Hospital, Darlinghurst, Australia; 3 St Vincent’s Clinical Campus, School of Clinical Medicine, Faculty of Medicine and Health, University of New South Wales, Sydney, Australia; 4 Clinical Diabetes, Appetite and Metabolism, Garvan Institute of Medical Research, Darlinghurst, Australia; 5 Endocrinology and Metabolism, National Health Insurance Ilsan Hospital, Goyang-shi, Gyeonggi-do, South Korea; 6 National Drug and Alcohol Research Centre, University of New South Wales, Sydney, Australia; 7 Department of Cardiology, Concord Repatriation General Hospital, Sydney, Australia; 8 Faculty of Medicine and Health, University of Sydney, Sydney, Australia; 9 Department of Clinical Pharmacology and Toxicology, St Vincent’s Hospital, Sydney, Australia

**Keywords:** Pharmacoepidemiology, Sodium-glucose co-transporter 2 inhibitors, Glucagon-like peptide-1 receptor agonists, Trends, Type 2 diabetes, Cardiovascular disease

## Abstract

**Purpose:**

To investigate trends in SGLT2i and GLP-1RA use in Australia in the era of increased evidence of their cardiovascular benefits.

**Methods:**

We used national dispensing claims for a 10% random sample of Australians to estimate the number of prevalent and new users (no dispensing in the prior year) of SGLT2i or GLP-1RA per month from January 2014 to July 2022. We assessed prescriber specialty and prior use of other antidiabetic and cardiovascular medicines as a proxy for evidence of type 2 diabetes (T2D) and cardiovascular conditions, respectively.

**Results:**

We found a large increase in the number of prevalent users (216-fold for SGLT2i; 11-fold for GLP-1RA); in July 2022 approximately 250,000 Australians were dispensed SGLT2i and 120,000 GLP-1RA. Most new users of SGLT2i or GLP-1RA had evidence of both T2D and cardiovascular conditions, although from 2022 onwards, approximately one in five new users of SGLT2i did not have T2D. The proportion of new users initiating SGLT2i by cardiologists increased after 2021, reaching 10.0% of initiations in July 2022. Among new users with evidence of cardiovascular conditions, empagliflozin was the most commonly prescribed SGLT2i, while dulaglutide or semaglutide was the most common GLP-1RA.

**Conclusion:**

SGLT2i and GLP-1RA use is increasing in Australia, particularly in populations with higher cardiovascular risk. The increased use of SGLT2i among people without evidence of T2D suggests that best-evidence medicines are adopted in Australia across specialties, aligning with new evidence and expanding indications.

**Supplementary Information:**

The online version contains supplementary material available at 10.1007/s00228-023-03539-8.

## Introduction

Sodium-glucose co-transporter 2 inhibitors (SGLT2i) and glucagon-like peptide-1 receptor agonists (GLP-1RA) are two classes of glucose-lowering medicines [1]. SGLT2i prevents the kidneys from reabsorbing glucose to the systemic circulation, resulting in urinary glucose excretion; GLP-1RA stimulates glucose-dependent insulin secretion and suppresses glucagon secretion, leading to lower plasma glucose levels [1]. Due to their ability to reduce blood glucose, SGLT2i and GLP-1RA have been used to treat type 2 diabetes (T2D) for over a decade [2, 3].

Since 2015, clinical trials have also shown these medicines significantly reduce cardiovascular events among patients with T2D and established cardiovascular disease (CVD) or high cardiovascular risk (Table S1). SGLT2i reduces hospitalisations for heart failure by 27–35% and major adverse cardiovascular events (MACE) in patients with T2D and at high cardiovascular risk by 14–20% [1]. Similarly, GLP-1RA reduces MACE by 12% among people with T2D [4]. Due to the cardiovascular benefits of SGLT2i and GLP-1RA, recent clinical guidelines for diabetes treatment recommend the use of SGLT2i or GLP-1RA for people with concomitant T2D and other cardiovascular risk factors [5, 6].

More recent trials have shown protective effects of SGLT2i in other populations, regardless of T2D: SGLT2i significantly decreases re-hospitalisations and cardiovascular deaths among people with heart failure [7], and reduces end-stage kidney disease and renal deaths among people with chronic kidney disease [8]. SGLT2i is now recommended for people with early-stage heart failure [5, 9] and people with chronic kidney disease, regardless of T2D [10]. The Australian Pharmaceutical Benefits Scheme (PBS) also expanded SGLT2i therapeutic indications to include heart failure and chronic kidney disease in January and September 2022, respectively (Table S2). GLP-1RA have also shown significant effects in reducing body weight in people with overweight or obesity [11, 12], but they have not been indicated for treating weight loss in the PBS.

Despite advances in knowledge about the broader cardiovascular benefits of SGLT2i and GLP-1RA, there is limited evidence on trends in the uptake of these therapies among different clinical populations [13] and prescribers [14, 15], particularly in Australia. Prior studies show that SGLT2i and GLP-1RA use among people with T2D have substantially increased worldwide [3]. However, the use of these therapies is still suboptimal in many jurisdictions. A multinational study showed that only 15% of people with T2D were prescribed SGLT2i or GLP-1RA as an add-on therapy between 2017 and 2019 [3], with similar rates observed in Australia during 2012–2017 [2]. As T2D is largely managed in primary care, general practitioners (GPs) prescribe most of the SGLT2i and GLP-1RA [14, 15]. However, GPs have reported the need for further education and engagement with endocrinologists in managing T2D [16]. The role cardiologists, renal physicians and other specialities, in prescribing these medicines is expected to increase with expanding therapeutic indications.

We therefore used dispensing data from a nationally representative sample of Australians in the period 2014–2022 to: 1) quantify trends in SGLT2i or GLP-1RA use in people with evidence of T2D and/or cardiovascular conditions, and by the specialty of prescriber; 2) quantify changes in characteristics of new users of SGLT2i or GLP-1RA; and 3) describe the uptake of specific SGLT2i and GLP-1RA medicines among new users with evidence of cardiovascular conditions.

## Methods

### Setting

In Australia, a national healthcare system provides subsidised prescription medicines to all citizens and eligible residents under the PBS [17]. People contribute a co-payment towards the cost of their medicines, which varies depending on their government entitlements. Concessional beneficiaries, such as pensioners, low-income earners and people with disability, pay a lower co-payment for medicines listed in the PBS than non-concessional beneficiaries [18, 19]. Medicines dispensed in the community, private hospitals and on discharge from public hospitals in most states are captured in the PBS, but not those dispensed privately or to public hospital inpatients [17].

### Data source

We used the dispensing records for a 10% random sample of PBS-eligible people [17]. The PBS 10% sample contains information about medicines dispensed (PBS item code, supply date), prescribers, and patients (year of birth, sex and year of death). We mapped PBS item codes to generic names and Anatomic Therapeutic Chemical (ATC) codes. To protect individual privacy, all dispensing dates are offset 14 days; the direction of the offset is the same for all records of each person.

### Medicines of interest

**Fig. 1:**
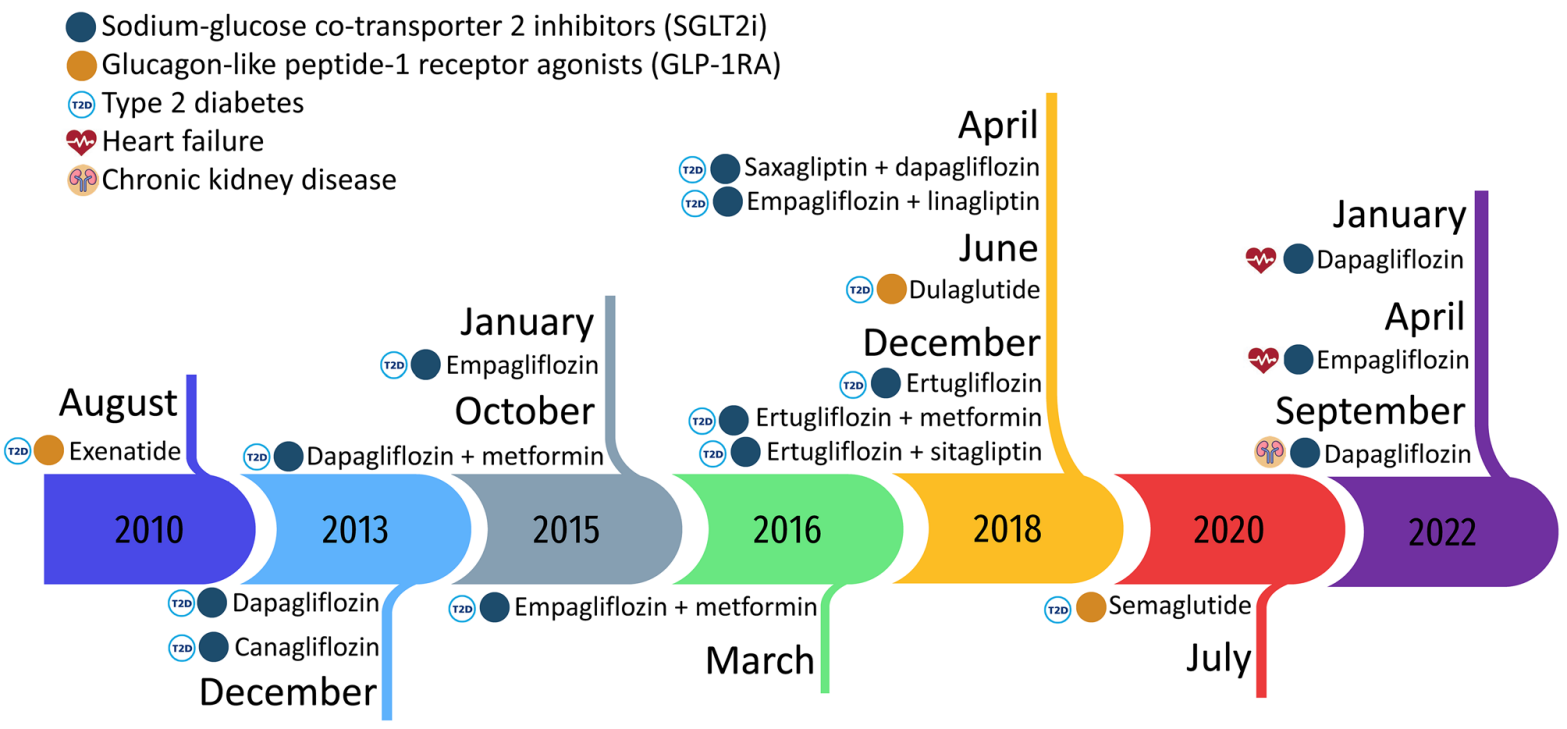
Timeline of SGLT2i and GLP-1RA listed in the Australian Pharmaceutical Benefits Scheme by indication. SGLT2i, Sodium-glucose co-transporter 2 inhibitors; GLP-1RA, Glucagon-like peptide-1 receptor agonists

In Australia, three GLP-1RA (exenatide, dulaglutide and semaglutide) and four SGLT2i medicines (canagliflozin, dapagliflozin, empagliflozin and ertugliflozin) have been PBS-listed for T2D since mid-2010 and late 2013, respectively. Since then, canagliflozin was delisted by the sponsor in 2015. The PBS-indications were expanded in dapagliflozin and empagliflozin for treating symptomatic heart failure, in January and April 2022 respectively (see details in Fig. 1 and Table S2). We identified the medicines of interest, other T2D medicines and cardiovascular medicines using PBS and ATC codes (Table S3).

### Study population

We included prevalent users, i.e. people who were dispensed SGLT2i or GLP-1RA, aged ≥ 18 years between January 2014 and July 2022. We examined each medicine class separately; prevalent users of both classes contributed to each analysis. We defined new users as no dispensing of medicines in that class in the prior year [20].

As a proxy for clinical indication of either T2D and/or cardiovascular conditions, we examined the use of other medicines to treat these conditions in the index dispensing or the prior 12 months. We defined prior use of other T2D medicines as any dispensing of a blood glucose lowering medicine other than SGLT2i or GLP-1RA. We considered the dispensing of insulin alone as indicative of type 1 diabetes. We defined prior cardiovascular medicine use as at least one dispensing of cardiovascular medicines used for the prevention or treatment of CVD (Table S3).

### Trends in SGLT2i or GLP-1RA use

We explored monthly trends in SGLT2i or GLP-1RA use, including the number of dispensings and the number of prevalent users of these medicines. Second, we reported the monthly number of new users of SGLT2i or GLP-1RA, including stratification by evidence of clinical conditions (T2D and/or cardiovascular conditions) and prescriber specialty.

### Characteristics of new users of SGLT2i or GLP-1RA

We reported the characteristics of new users grouped by two-year intervals (2014–2015, 2016–2017, 2018–2019, 2020–2021) and January-July 2022. New users could contribute to multiple time intervals if they met the definition of initiation in each.

We reported characteristics at the date of initiation, including age, sex, PBS concessional status and specialty of prescriber (endocrinologist, cardiologist, nephrologist and GP/others/unknown). We also identified morbidities using the Rx-Risk Comorbidity Index, which maps 46 morbidities to ATC codes in a 12-month look back period (i.e. 360 days before and including the index dispensing) and calculated a total weighted comorbidity score [21]. We also reported selected morbidities (arrhythmia, congestive heart failure, hyperlipidaemia, hypertension, ischaemic heart disease: angina, ischaemic heart disease: hypertension, renal disease) and selected medicine groups (anticoagulants, antiplatelets) (Table S3).

### SGLT2i or GLP-1RA medicines initiated among new users with evidence of cardiovascular conditions

We selected a subgroup of new users who had evidence of cardiovascular conditions to investigate specific medicines initiated, reporting the monthly percentage of new users initiating each SGLT2i and GLP-1RA medicine.

We performed all analyses using SAS version 9.4 (SAS Institute Inc., Cary, NC, USA) and produced figures using R version 4.2.3 (R Core Team 2017, Vienna, Austria).

## Results

### Trends in SGLT2i or GLP-1RA use

**Fig. 2:**
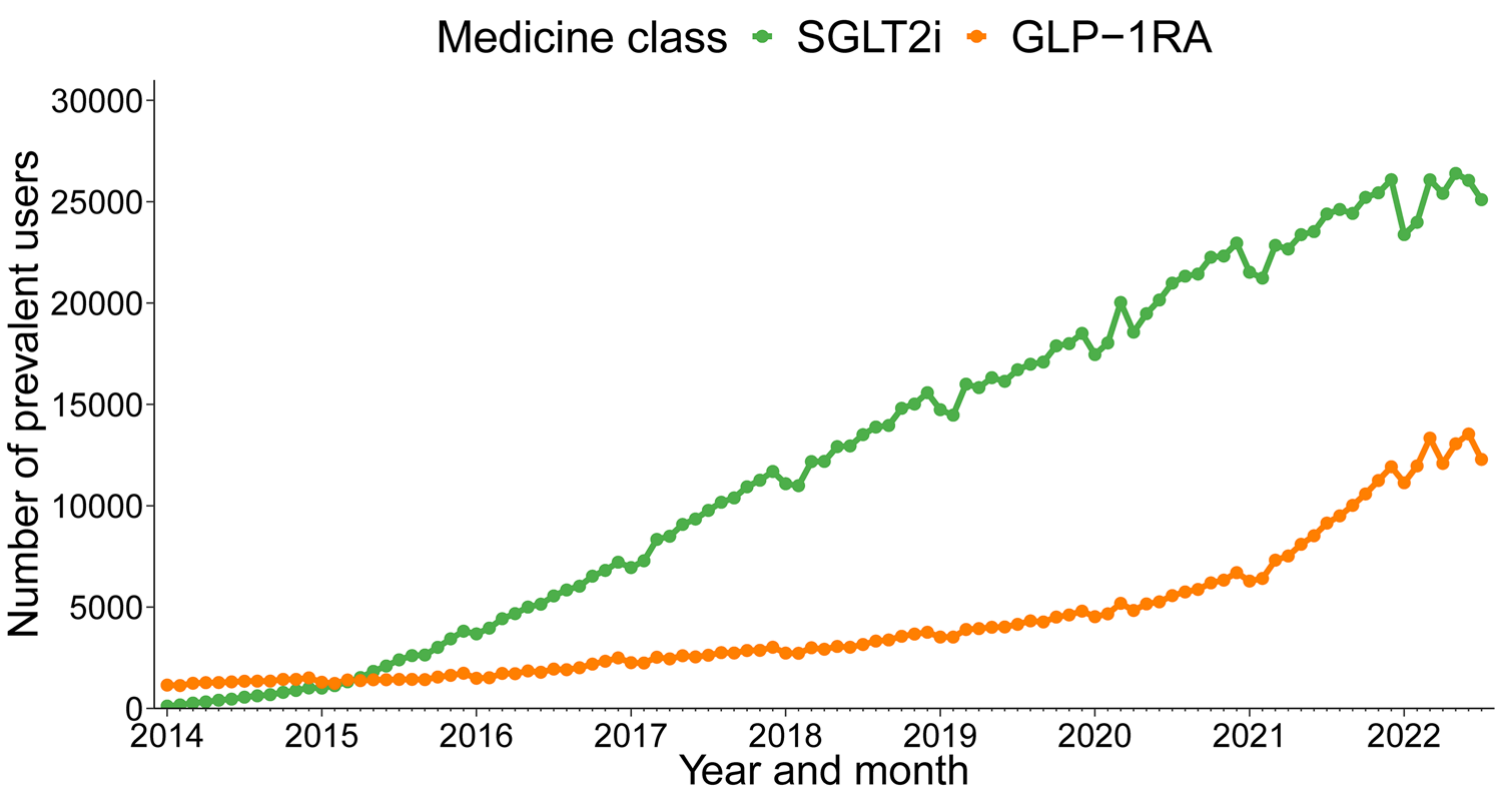
Monthly trends in the number of prevalent users, January 2014-July 2022. SGLT2i, Sodium-glucose co-transporter 2 inhibitors; GLP-1RA, Glucagon-like peptide-1 receptor agonists

**Fig. 3:**
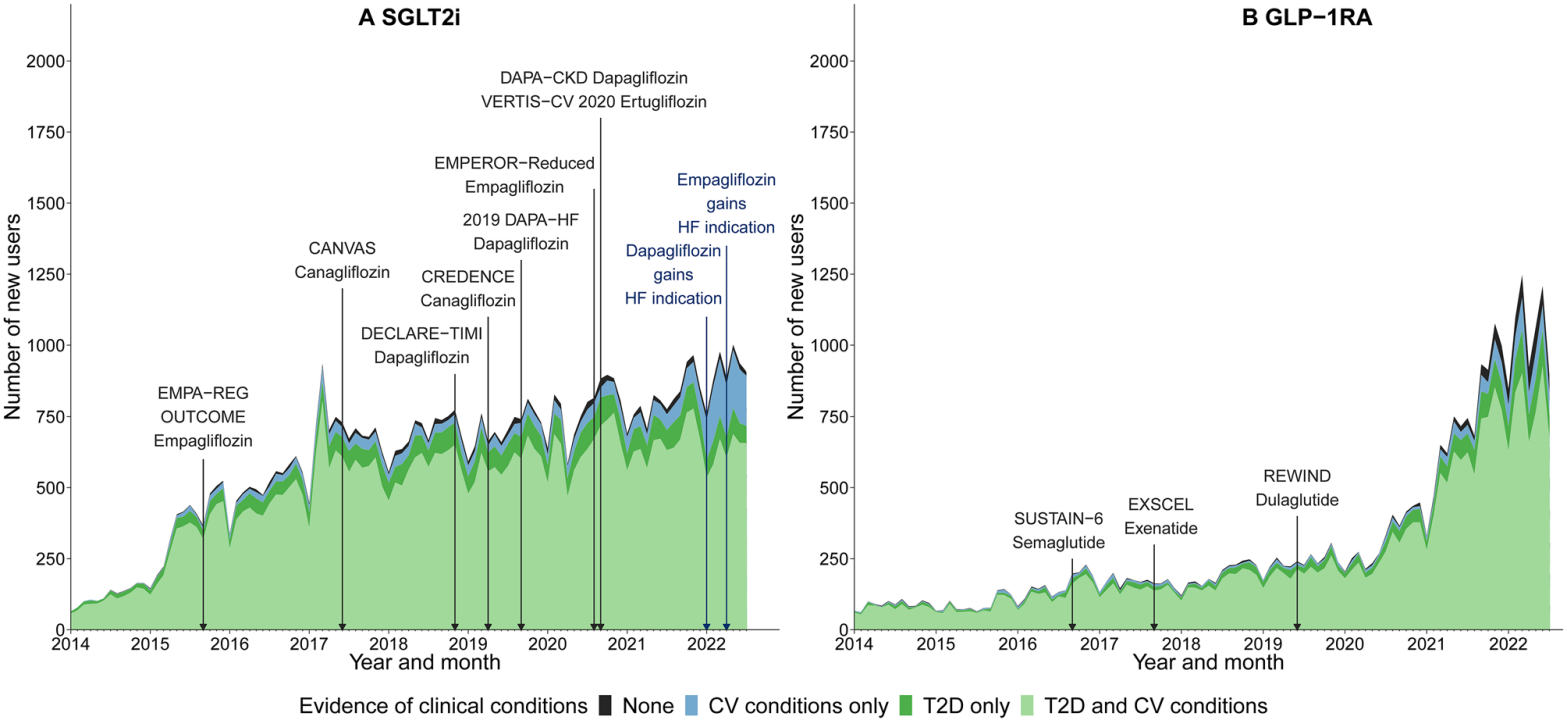
Monthly trends in the number of new users by evidence of clinical conditions. SGLT2i, Sodium-glucose co-transporter 2 inhibitors; GLP-1RA, Glucagon-like peptide-1 receptor agonists; CV, Cardiovascular; T2D, Type 2 diabetes. See Table S1 for the details of each clinical trial

**Fig. 4:**
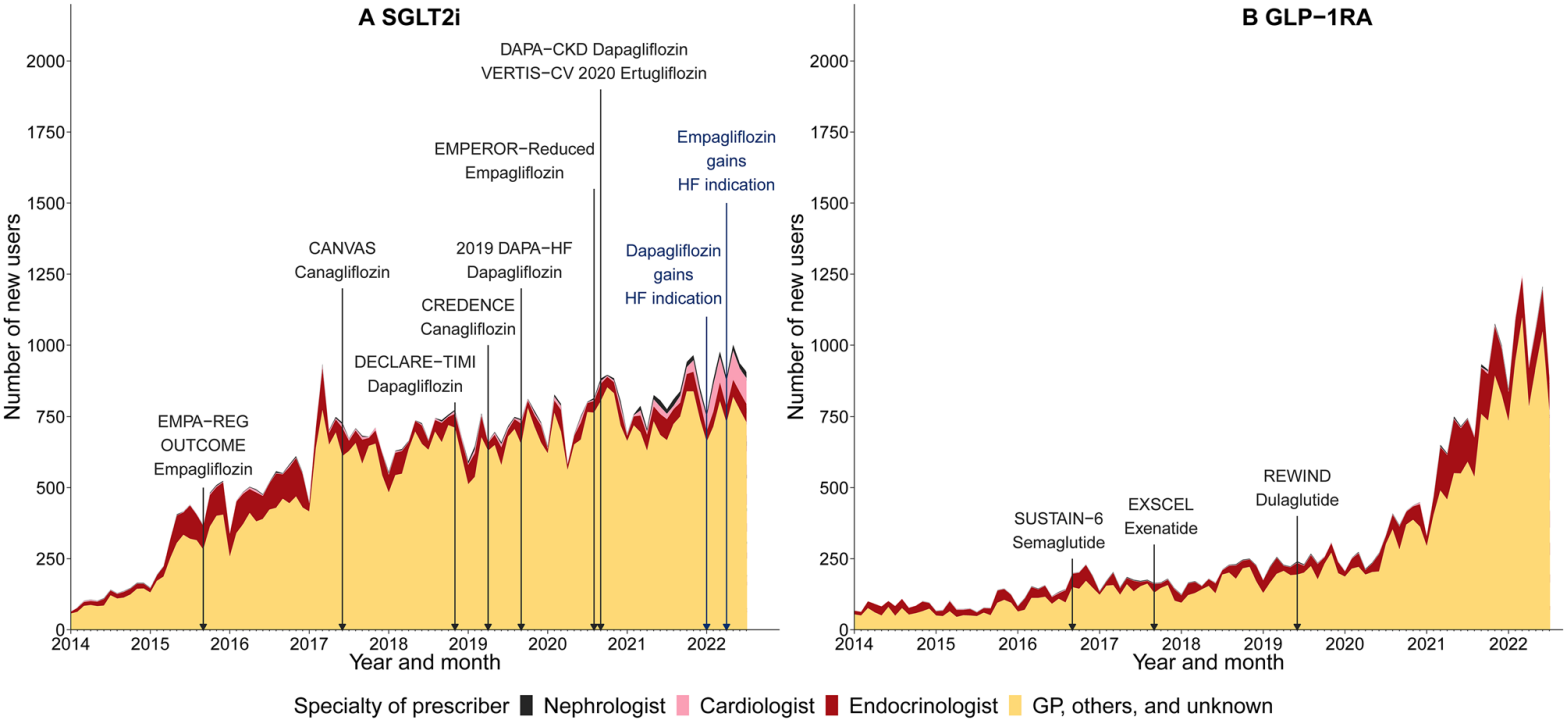
Monthly trends in the number of new users by specialty of prescriber. SGLT2i, Sodium-glucose co-transporter 2 inhibitors; GLP-1RA, Glucagon-like peptide-1 receptor agonists; GP, General practitioner. See Table S1 for the details of each clinical trial.

We identified a total of 67,207 prevalent users of either SGLT2i (n = 56,117) and/or GLP1-RA (n = 27,988) during the study period. The number of prevalent users of SGLT2i substantially increased, from 116 people in January 2014 up to 25,099 in July 2022 (Fig. 2). The number of prevalent users of GLP-1RA increased gradually from 2014 to 2020, and rapidly from 2021 to 2022; an increase from 1,153 people in January 2014 up to 12,286 in July 2022. We observed similar trends in the monthly number of new users (SGLT2i: from 65 to 907 people; GLP-1RA: from 67 to 896 people) (Figs. 3 and 4) and the total monthly number of dispensings (SGLT2i: from 138 to 28,654 dispensings; GLP-1RA: from 1,289 to 14,914 dispensings) (Figs. S1 and S2).

Most new users of SGLT2i or GLP-1RA had evidence of T2D and cardiovascular conditions (Fig. 3). However, we observed a rapid increase after 2021 in new users of either class of medicine who had evidence of cardiovascular conditions but not T2D. This increase was more pronounced for SGLT2i (from 1.5% of new users in January 2014 to 19.5% in July 2022) than GLP-1RA (from 4.5% to 8.0% respectively) (Fig. 3). We also observed a large increase in the number of dispensings among those with evidence of cardiovascular conditions but not T2D (from 1.4% to 17.3% for SGLT2i; from 1.4% to 4.8% for GLP-1RA) (Fig. S1).

A relatively small proportion of new users of SGLT2i and GLP-1RA were prescribed these medicines by cardiologists, endocrinologists and nephrologists, with the majority prescribed by GPs or other medical specialists (Fig. 4). However, the number of new users initiating either medicine class by these specialist groups increased over time, particularly SGLT2i prescribed by cardiologists − which increased after 2021 and represented 10.0% of new users in July 2022 (Fig. 4). We also observed most medicines were prescribed by clinicians other than cardiologists, endocrinologists and nephrologists; although there was an increased proportion of dispensings prescribed by cardiologists for SGLT2i (representing 1.8% of dispensings) but not for GLP-1RA (Fig. S2).

### Characteristics of new users of SGLT2i or GLP-1RA

Compared to earlier in the study period, new users of SGLT2i in more recent years tended to be older, with a slightly higher percentage of males and evidence of treatment for heart failure, and lower percentage of concessional beneficiaries and evidence of T2D (Table 1). In 2022, 53.5% of new users of SGLT2i were aged 65 years and older, 61.6% were male, 25.0% had evidence of treatment for heart failure, 57.6% were concessional beneficiaries, and 77.4% had evidence of T2D. Compared to earlier in the study period, new users of GLP-1RA also tended to be older, with a higher proportion of females and people with evidence of T2D (Table 2).

**Table 1:** Characteristics of new users of SGLT2i

**Characteristics**	**2014–2015**	**2016–2017**	**2018–2019**	**2020–2021**	**Jan-Jul 2022**
Number of people, n (%)	5,839 (100.0)	14,283 (100.0)	16,467 (100.0)	18,639 (100.0)	6,352 (100.0)
Age, years, median (IQR)	61 (53–68)	62 (53–69)	62 (53–70)	63 (54–72)	66 (56–75)
Age group, years, n (%)					
18–49	1,064 (18.2)	2,391 (16.7)	2,847 (17.3)	3,232 (17.3)	895 (14.1)
50–64	2,626 (45.0)	6,053 (42.4)	6,618 (40.2)	6,816 (36.6)	2,061 (32.4)
65–74	1,642 (28.1)	4,175 (29.2)	4,679 (28.4)	5,401 (29.0)	1,804 (28.4)
75–84	457 (7.8)	1,496 (10.5)	2,022 (12.3)	2,714 (14.6)	1,275 (20.1)
≥ 85	50 (0.9)	168 (1.2)	301 (1.8)	476 (2.6)	317 (5.0)
Sex, n (%)					
Women	2,528 (43.3)	5,826 (40.8)	6,516 (39.6)	7,162 (38.4)	2,437 (38.4)
Men	3,311 (56.7)	8,457 (59.2)	9,951 (60.4)	11,477 (61.6)	3,915 (61.6)
Concessional beneficiaries, n (%)	3,479 (59.6)	8,412 (58.9)	9,339 (56.7)	10,755 (57.7)	3,659 (57.6)
Number of morbidities, median (IQR)	5 (3–6)	5 (3–6)	4 (3–6)	4 (3–6)	5 (3–7)
Number of morbidities, n (%)					
0–2	899 (15.4)	2,312 (16.2)	2,985 (18.1)	3,579 (19.2)	1,056 (16.6)
3–5	2,784 (47.7)	6,720 (47.0)	7,796 (47.3)	8,625 (46.3)	2,800 (44.1)
> 5	2,156 (36.9)	5,251 (36.8)	5,686 (34.5)	6,435 (34.5)	2,496 (39.3)
History of medicine use and morbidities^*^, n (%)					
Type 2 diabetes	5,625 (96.3)	13,389 (93.7)	15,336 (93.1)	17,065 (91.6)	4,915 (77.4)
Cardiovascular conditions	5,238 (89.7)	12,709 (89.0)	14,481 (87.9)	16,335 (87.6)	5,678 (89.4)
Anticoagulants	375 (6.4)	1,207 (8.5)	1,538 (9.3)	2,025 (10.9)	1,156 (18.2)
Antiplatelets	1,040 (17.8)	1,966 (13.8)	1,823 (11.1)	1,883 (10.1)	687 (10.8)
Arrhythmia	189 (3.2)	608 (4.3)	705 (4.3)	854 (4.6)	523 (8.2)
Congestive heart failure	437 (7.5)	1,243 (8.7)	1,554 (9.4)	2,121 (11.4)	1,588 (25.0)
Hyperlipidaemia	4,473 (76.6)	10,715 (75.0)	12,034 (73.1)	13,482 (72.3)	4,567 (71.9)
Hypertension	2,687 (46.0)	6,261 (43.8)	6,722 (40.8)	7,351 (39.4)	2,651 (41.7)
Ischaemic heart disease: angina	366 (6.3)	989 (6.9)	1,067 (6.5)	1,227 (6.6)	479 (7.5)
Ischaemic heart disease: hypertension	2,419 (41.4)	6,086 (42.6)	6,765 (41.1)	7,549 (40.5)	2,407 (37.9)
Renal disease	16 (0.3)	35 (0.2)	54 (0.3)	78 (0.4)	52 (0.8)

People may be included in multiple time intervals

*SGLT2i* Sodium-glucose co-transporter 2 inhibitors, *IQR* Interquartile range

^*^Dispensed medicines in the year prior including the date of initiation

**Table 2:** Characteristics of new users of GLP-1RA

**Characteristics**	**2014–2015**	**2016–2017**	**2018–2019**	**2020–2021**	**Jan-Jul 2022**
Number of people, n (%)	2,086 (100.0)	3,793 (100.0)	5,095 (100.0)	12,488 (100.0)	7,289 (100.0)
Age, years, median (IQR)	59 (50–66)	60 (51–68)	60 (51–69)	61 (51–69)	60 (50–69)
Age group, years, n (%)					
18–49	474 (22.7)	834 (22.0)	1,064 (20.9)	2,594 (20.8)	1,676 (23.0)
50–64	990 (47.5)	1,659 (43.7)	2,091 (41.0)	5,070 (40.6)	2,917 (40.0)
65–74	494 (23.7)	973 (25.7)	1,399 (27.5)	3,344 (26.8)	1,841 (25.3)
75–84	113 (5.4)	295 (7.8)	470 (9.2)	1,297 (10.4)	752 (10.3)
≥ 85	15 (0.7)	32 (0.8)	71 (1.4)	183 (1.5)	103 (1.4)
Sex, n (%)					
Women	1,022 (49.0)	1,909 (50.3)	2,588 (50.8)	6,209 (49.7)	3,884 (53.3)
Men	1,064 (51.0)	1,884 (49.7)	2,507 (49.2)	6,279 (50.3)	3,405 (46.7)
Concessional beneficiaries, n (%)	1,212 (58.1)	2,390 (63.0)	3,079 (60.4)	7,086 (56.7)	3,817 (52.4)
Number of morbidities, median (IQR)	5 (3–7)	5 (4–7)	5 (3–7)	5 (3–7)	5 (3–6)
Number of morbidities, n (%)					
0–2	262 (12.6)	474 (12.5)	693 (13.6)	1,862 (14.9)	1,279 (17.5)
3–5	927 (44.4)	1,589 (41.9)	2,243 (44.0)	5,694 (45.6)	3,349 (45.9)
> 5	897 (43.0)	1,730 (45.6)	2,159 (42.4)	4,932 (39.5)	2,661 (36.5)
History of medicine use and morbidities^*^, n (%)					
Type 2 diabetes	1,995 (95.6)	3,561 (93.9)	4,859 (95.4)	11,547 (92.5)	6,180 (84.8)
Cardiovascular conditions	1,896 (90.9)	3,411 (89.9)	4,526 (88.8)	10,888 (87.2)	5,984 (82.1)
Anticoagulants	168 (8.1)	364 (9.6)	474 (9.3)	1,201 (9.6)	702 (9.6)
Antiplatelets	396 (19.0)	578 (15.2)	562 (11.0)	1,231 (9.9)	556 (7.6)
Arrhythmia	80 (3.8)	134 (3.5)	195 (3.8)	414 (3.3)	240 (3.3)
Congestive heart failure	208 (10.0)	448 (11.8)	526 (10.3)	1,286 (10.3)	679 (9.3)
Hyperlipidaemia	1,606 (77.0)	2,850 (75.1)	3,823 (75.0)	9,036 (72.4)	4,762 (65.3)
Hypertension	1,067 (51.2)	1,814 (47.8)	2,225 (43.7)	5,148 (41.2)	2,810 (38.6)
Ischaemic heart disease: angina	146 (7.0)	277 (7.3)	341 (6.7)	730 (5.8)	332 (4.6)
Ischaemic heart disease: hypertension	917 (44.0)	1,622 (42.8)	2,146 (42.1)	5,078 (40.7)	2,725 (37.4)
Renal disease	7 (0.3)	23 (0.6)	32 (0.6)	91 (0.7)	44 (0.6)

People may be included in multiple time intervals

*GLP-1RA* Glucagon-like peptide-1 receptor agonists, *IQR* Interquartile range

*Dispensed medicines in the year prior including the date of initiation

### SGLT2i or GLP-1RA medicines initiated among new users with evidence of cardiovascular conditions

**Fig. 5:**
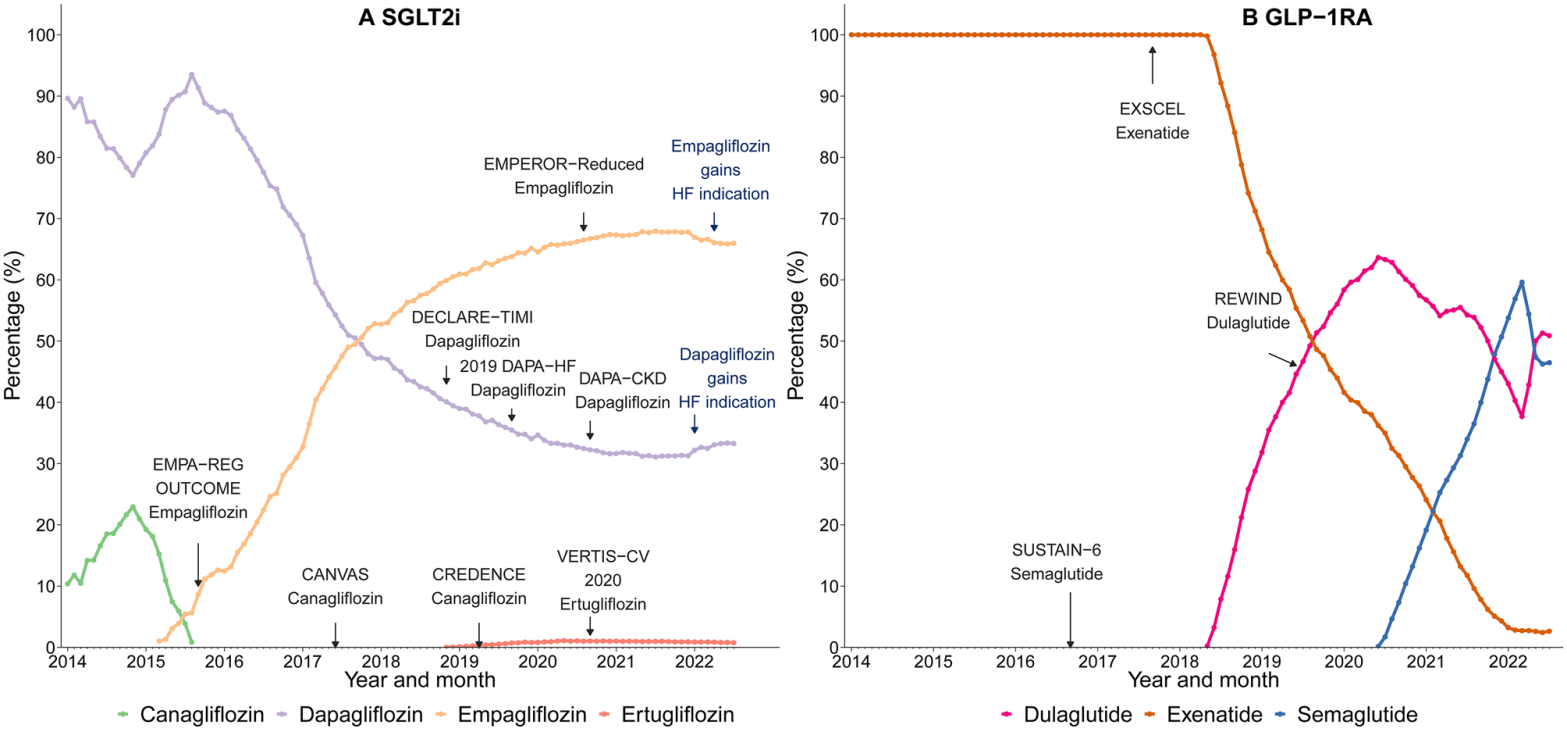
Percentage of new users with evidence of cardiovascular conditions initiating specific medicines. SGLT2i, Sodium-glucose co-transporter 2 inhibitors; GLP-1RA, Glucagon-like peptide-1 receptor agonists. See Table S1 for the details of each clinical trial

We observed substantial changes in the medicines initiated among new users over time with evidence of cardiovascular conditions (Fig. 5). For SGLT2i, the percentage of new users initiating dapagliflozin decreased, whilst initiation on empagliflozin increased: in July 2022, most people were initiated on empagliflozin (66.0%). For GLP-1RA, the percentage of new users who initiated on exenatide decreased when other GLP-1RA medicines (dulaglutide and semaglutide) were listed on the PBS. Similarly, the percentage of new users initiating dulaglutide decreased when semaglutide was introduced, although increased rapidly from April 2022. In July 2022, almost all new users of GLP-1RA initiated dulaglutide (50.9%) or semaglutide (46.5%).

## Discussion

Our study provides a contemporary perspective on SGLT2i and GLP-1RA use in Australia in the context of increasing clinical evidence of cardiovascular benefits and expanding therapeutic indications for SGLT2i medicines for heart failure. Most new users of SGLT2i and GLP-1RA had evidence of treatment for CVD or CVD risk factors, including T2D. We also observed a rapid increase in the use of SGLT2i among those with evidence of cardiovascular conditions, but not T2D, as well as a rapid increase in prescribing by cardiologists, consistent with the expansion of indications for SGLT2i to include heart failure. In addition, empagliflozin became the most prescribed SGLT2i and dulaglutide or semaglutide the most prescribed GLP-1RA. Accordingly, new users of SGLT2i in 2022 tended to be older, more likely to be male, and have evidence of treatment for heart failure, but not T2D, than in earlier years. Changes in the characteristics of new users of GLP-1RA were less pronounced.

Overall, the increasing trends in SGLT2i and GLP-1RA use suggest that clinicians are applying the best available evidence. While the trends of increasing use found in our study are similar to those seen in Australian studies in earlier years [2, 22, 23], there was a much larger increase from 2016, coinciding with the first trials among people with T2D showing cardiovascular benefits of SGLT2i and GLP-1RA in late 2015 [1]. Moreover, our findings are similar to other countries which also reported increased use of these medicines over time, such as the United Kingdom [2, 24], Denmark [25], Canada [2, 26] and the United States (US) [14, 15, 27].

Notably, the majority of SGLT2i were prescribed by clinicians other than cardiologists, endocrinologists and nephrologists. This is also consistent with prescribing patterns in the US [14, 15] and Denmark [25]. The small percentage of SGLT2i and GLP-1RA prescribed by cardiologists prior to 2022 possibly relates to cardiologists’ lack of confidence in prescribing those agents which are perceived as diabetes drugs [28]. However, we observed a rapid increase in SGLT2i use since the addition of heart failure to PBS indications, alongside an increase in prescribing by cardiologists, and use of these medicines among people with possible evidence of heart failure. The increased prescribing by cardiologists likely also reflects recommendations of using SGLT2i in recent guidelines from cardiac societies [5, 9]. We also observed few SGLT2i dispensings prescribed by nephrologists, which is likely due to the absence of chronic kidney disease as a PBS indication for SGLT2i during the study period. However, no such rapid increase was observed for GLP-1RA, possibly due to the lack of evidence of cardiovascular benefits and reimbursement for people without T2D.

Encouragingly, we found that medicines with higher cardiovascular benefits such as empagliflozin [29] and dulaglutide or semaglutide [30, 31] were the most commonly initiated in the subgroup of new users with evidence of cardiovascular conditions. This was similar to findings among people with T2D with a history of myocardial infarction, stroke or heart failure in the US [15]. This indicates clinicians are up to date with guidelines and clinical evidence of the cardiovascular benefits of these medicines.

The lower proportion of new users with evidence of T2D in recent years shows a tendency for both SGLT2i and GLP-1RA to be used for indications beyond T2D. This was particularly interesting for GLP-1RA, as PBS indications in Australia currently do not support the use of GLP-1RA beyond T2D – inferring the possibility of off-label use for other indications such as weight loss. The higher percentage of female users of GLP-1RA in recent years could similarly be related to the clinical evidence of weight loss of GLP-1RA published in 2021 [11].

Given we used a 10% representative sample of the population, we estimate approximately 670,000 Australians received SGLT2i and/or GLP-1RA during the study period, the majority of whom had evidence of T2D or cardiovascular conditions. Approximately 1.16 million Australians have been diagnosed with T2D, with 60,000 new T2D diagnoses every year [32]. Around 1.15 million Australians are estimated to have one or more self-reported cardiovascular conditions, with over 100,000 having heart failure, and over 250,000 having concurrent diabetes [33]. While not all these people have an indication for the use of SGLT2i or GLP-1RA, based on these estimates there is potential for further optimising the use of SGLT2i and GLP-1RA relative to the large number of Australians with T2D and CVD (including heart failure) [33].

As the body of evidence of benefits of SGLT2i and GLP-1RA continues to grow and their indications expand (e.g. SGLT2i for chronic kidney disease [8, 34–36] and GLP-1RA for weight loss [11, 12]), it is important to continue monitoring patterns of use of these medicines and understanding barriers for their uptake. More research is needed to explore what factors are associated with the use of SGLT2i and GLP-1RA, such as use in clinically indicated populations and across different socio-demographic groups, to provide evidence for further strategies for improving SGLT2i and GLP-1RA use.

Our study has some limitations. First, PBS data do not capture private and public hospital inpatient prescriptions (e.g. for medicines not listed in the PBS such as liraglutide or PBS-listed medicines privately purchased), and so may underestimate SGLT2i and GLP-1RA use. Second, since no information regarding indication for prescribing is available in the PBS data, we derived proxies from dispensing of other medicines, which may be subject to misclassification. Third, there were shortages of some medicines of interest during the study period (Table S2), which may have affected usage patterns. Fourth, given the study period included the COVID-19 pandemic, changes in patient access to health services and prescriber access to educational activities may have affected SGLT2i and GLP-1RA use. However, this may not have a great impact on patient access to health services given there was no reduction in the number of Medicare Benefits Scheme claims (e.g. GP attendance) and PBS dispensings during the pandemic [37].

## Conclusion

SGLT2i and GLP-1RA use increased substantially in accordance with the increasing evidence of cardiovascular benefits. The use of SGLT2i and GLP-1RA prescribed by cardiologists increased over time, and rapidly increased from 2022 when the therapeutic indication for heart failure was included in the PBS. In recent years, medicines with higher cardiovascular benefits such as empagliflozin and semaglutide accounted for a large percentage of new users with evidence of cardiovascular conditions. This study shows the diffusion of medicines aligns with new evidence and indications and suggests there is potential to further optimise the use of these evidence-based therapies in Australia.

## Supplementary Information

Below is the link to the electronic supplementary material.

Supplementary file1 (DOCX 418 KB)

## Data Availability

The PBS 10% sample was provided by the Australian Government Services Australia under licence. Access to these data by other individuals or authorities is not permitted without the express permission of the approving human research ethics committees and data custodians.

## References

[1] Brown E, Heerspink HJL, Cuthbertson DJ, Wilding JPH (2021). SGLT2 inhibitors and GLP-1 receptor agonists: established and emerging indications. The Lancet.

[2] Greiver M, Havard A, Bowles JKF, Kalia S, Chen T, Aliarzadeh B (2021). Trends in diabetes medication use in Australia, Canada, England, and Scotland: a repeated cross-sectional analysis in primary care. Br J Gen Pract.

[3] Arnold SV, Tang F, Cooper A, Chen H, Gomes MB, Rathmann W (2022). Global use of SGLT2 inhibitors and GLP-1 receptor agonists in type 2 diabetes. Results from DISCOVER BMC Endocr Disord.

[4] Kristensen SL, Rørth R, Jhund PS, Docherty KF, Sattar N, Preiss D (2019). Cardiovascular, mortality, and kidney outcomes with GLP-1 receptor agonists in patients with type 2 diabetes: a systematic review and meta-analysis of cardiovascular outcome trials. Lancet Diabetes Endocrinol.

[5] Seferović PM, Fragasso G, Petrie M, Mullens W, Ferrari R, Thum T et al (2020) Sodium–glucose co-transporter 2 inhibitors in heart failure: beyond glycaemic control. A position paper of the Heart Failure Association of the European Society of Cardiology. Eur J Heart Fail 22(9):1495–1503 10.1002/ejhf.1954 32618086

[6] Khan MS, Fonarow GC, McGuire DK, Hernandez AF, Vaduganathan M, Rosenstock J (2020). Glucagon-Like Peptide 1 Receptor Agonists and Heart Failure: The Need for Further Evidence Generation and Practice Guidelines Optimization. Circulation.

[7] Zannad F, Ferreira JP, Pocock SJ, Anker SD, Butler J, Filippatos G (2020). SGLT2 inhibitors in patients with heart failure with reduced ejection fraction: a meta-analysis of the EMPEROR-Reduced and DAPA-HF trials. The Lancet.

[8] Heerspink HJL, Stefánsson BV, Correa-Rotter R, Chertow GM, Greene T, Hou FF (2020). Dapagliflozin in Patients with Chronic Kidney Disease. N Engl J Med.

[9] Atherton JJ, Sindone A, De Pasquale CG, Driscoll A, MacDonald PS, Hopper I (2018). National Heart Foundation of Australia and Cardiac Society of Australia and New Zealand: Guidelines for the Prevention, Detection, and Management of Heart Failure in Australia 2018. Heart Lung Circ.

[10] UK Kidney Association (2021) UK Kidney Association Clinical Practice Guideline: Sodium-Glucose Co-transporter-2 (SGLT-2) Inhibition in Adults with Kidney Disease. https://ukkidney.org/sites/renal.org/files/UKKA%20guideline_SGLT2i%20in%20adults%20with%20kidney%20disease%20v1%2020.10.21.pdf

[11] Wilding JPH, Batterham RL, Calanna S, Davies M, Van Gaal LF, Lingvay I (2021). Once-Weekly Semaglutide in Adults with Overweight or Obesity. N Engl J Med.

[12] Garvey WT, Batterham RL, Bhatta M, Buscemi S, Christensen LN, Frias JP (2022). Two-year effects of semaglutide in adults with overweight or obesity: the STEP 5 trial. Nature Med.

[13] Zhuo M, Li J, Buckley LF, Tummalapalli SL, Mount DB, Steele DJR et al (2022) Prescribing Patterns of Sodium-Glucose Cotransporter-2 Inhibitors in Patients with CKD: A Cross-Sectional Registry Analysis. Kidney360 3(3):455–464 10.34067/KID.0007862021 PMC9034822 35582176

[14] Adhikari R, Jha K, Dardari Z, Heyward J, Blumenthal RS, Eckel RH (2022). National Trends in Use of Sodium-Glucose Cotransporter-2 Inhibitors and Glucagon-like Peptide-1 Receptor Agonists by Cardiologists and Other Specialties, 2015 to 2020. J Am Heart Assoc.

[15] Dave CV, Schneeweiss S, Wexler DJ, Brill G, Patorno E (2020). Trends in Clinical Characteristics and Prescribing Preferences for SGLT2 Inhibitors and GLP-1 Receptor Agonists, 2013–2018. Diabetes Care.

[16] Thepwongsa I, Kirby C, Paul C, Piterman L (2014). Management of type 2 diabetes: Australian rural and remote general practitioners' knowledge, attitudes, and practices. Rural Remote Health.

[17] Mellish L, Karanges EA, Litchfield MJ, Schaffer AL, Blanch B, Daniels BJ, Segrave A, Pearson SA (2015). The Australian Pharmaceutical Benefits Scheme data collection: a practical guide for researchers. BMC Res Notes.

[18] Australian Government Department of Healh and Aged Care (2022) Pharmaceutical Benefits Scheme - 5. The Safety Net Scheme. https://www.pbs.gov.au/info/healthpro/explanatory-notes/section1/Section_1_5_Explanatory_Notes

[19] Australian Government Department of Healh and Aged Care (2022) Pharmaceutical Benefits Scheme - Fees, Patient Contributions and Safety Net Thresholds. https://www.pbs.gov.au/info/healthpro/explanatory-notes/front/fee

[20] de Oliveira CJ, Gillies MB, Schaffer AL, Peiris D, Zoega H, Pearson SA (2022). Changes in antidepressant use in Australia: A nationwide analysis (2015–2021). Aust N Z J Psychiatry.

[21] Pratt NL, Kerr M, Barratt JD, Kemp-Casey A, Kalisch Ellett LM, Ramsay E (2018). The validity of the Rx-Risk Comorbidity Index using medicines mapped to the Anatomical Therapeutic Chemical (ATC) Classification System. BMJ Open.

[22] Morton JI, Ilomӓki J, Magliano DJ, Shaw JE (2021). The association of socioeconomic disadvantage and remoteness with receipt of type 2 diabetes medications in Australia: a nationwide registry study. Diabetologia.

[23] Morton JI, Ilomӓki J, Magliano DJ, Shaw JE (2022). Persistent disparities in diabetes medication receipt by socio-economic disadvantage in Australia. Diabet Med.

[24] Farmer RE, Beard I, Raza SI, Gollop ND, Patel N, Tebboth A (2021). Prescribing in Type 2 Diabetes Patients With and Without Cardiovascular Disease History: A Descriptive Analysis in the UK CPRD. Clin Ther.

[25] Pottegård A, Andersen JH, Søndergaard J, Thomsen RW, Vilsbøll T (2023) Changes in the use of glucose-lowering drugs: A Danish nationwide study. Diabetes Obes Metab 1–9 10.1111/dom.14947 36514856

[26] Fralick M, Martins D, Tadrous M, Gomes T (2022). Nationwide Trends in Dispensing of Sodium Glucose Cotransporter 2 Inhibitors. Can J Hosp Pharm.

[27] Williams BA, Brady JP, Voyce S, Kumar N, Paprocki Y, Rajpura J (2022). Changes over time in the cardiovascular risk profile of type 2 diabetes from 2007 to 2020: A community-based study. Diabetes Obes Metab.

[28] Milder TY, Stocker SL, Baysari M, Day RO, Greenfield JR (2021). Prescribing of SGLT2 inhibitors in primary care: A qualitative study of General Practitioners and Endocrinologists. Diabetes Res Clin Pract.

[29] Packer M, Anker SD, Butler J, Filippatos G, Pocock SJ, Carson P (2020). Cardiovascular and Renal Outcomes with Empagliflozin in Heart Failure. N Engl J Med.

[30] Marso SP, Bain SC, Consoli A, Eliaschewitz FG, Jódar E, Leiter LA (2016). Semaglutide and Cardiovascular Outcomes in Patients with Type 2 Diabetes. N Engl J Med.

[31] Gerstein HC, Colhoun HM, Dagenais GR, Diaz R, Lakshmanan M, Pais P (2019). Dulaglutide and cardiovascular outcomes in type 2 diabetes (REWIND): a double-blind, randomised placebo-controlled trial. The Lancet.

[32] Australian Institute of Health and Welfare (2022) Diabetes - Australian Facts. https://www.aihw.gov.au/reports/diabetes/diabetes/contents/about

[33] Australian Institute of Health and Welfare (2021) Heart, Stroke & Vascular Diseases - Australian Facts. https://www.aihw.gov.au/reports/heart-stroke-vascular-diseases/hsvd-facts/contents/risk-factors/multiple-risk-factors

[34] Neal B, Perkovic V, Mahaffey KW, de Zeeuw D, Fulcher G, Erondu N (2017). Canagliflozin and Cardiovascular and Renal Events in Type 2 Diabetes. N Engl J Med.

[35] Gerstein HC, Colhoun HM, Dagenais GR, Diaz R, Lakshmanan M, Pais P (2019). Dulaglutide and renal outcomes in type 2 diabetes: an exploratory analysis of the REWIND randomised, placebo-controlled trial. The Lancet.

[36] Carvalho PEP, Veiga TMA, Simões ESAC, Gewehr DM, Dagostin CS, Fernandes A et al (2023) Cardiovascular and renal effects of SGLT2 inhibitor initiation in acute heart failure: a meta-analysis of randomized controlled trials. Clin Res Cardiol:1–12. Epub ahead of print 10.1007/s00392-022-02148-2 PMC9807098 36592186

[37] Australian Institute of Health and Welfare (2022) Impacts of COVID-19 on Medicare Benefits Scheme and Pharmaceutical Benefits Scheme: quarterly data. https://www.aihw.gov.au/reports/health-care-quality-performance/impacts-of-covid19-mbs-pbs-quarterly-data/contents/about

