# Supplementary material for: Trends in use of sodium-glucose co-transporter 2 inhibitors (SGLT2i) and glucagon-like peptide-1 receptor agonists (GLP-1RA) in Australia in the era of increased evidence of their cardiovascular benefits (2014–2022)
Source: Eur J Clin Pharmacol. 2023 Jul 14;79(9):1239–48. doi: 10.1007/s00228-023-03539-8 (PMC10427543; doi:10.1007/s00228-023-03539-8)
Supplement: Supplementary file 1 — Supplementary file1 (DOCX 418 KB) [file 228_2023_3539_MOESM1_ESM.docx]

**Fig. S1** Monthly trends in the number of dispensings by evidence of clinical conditions

**
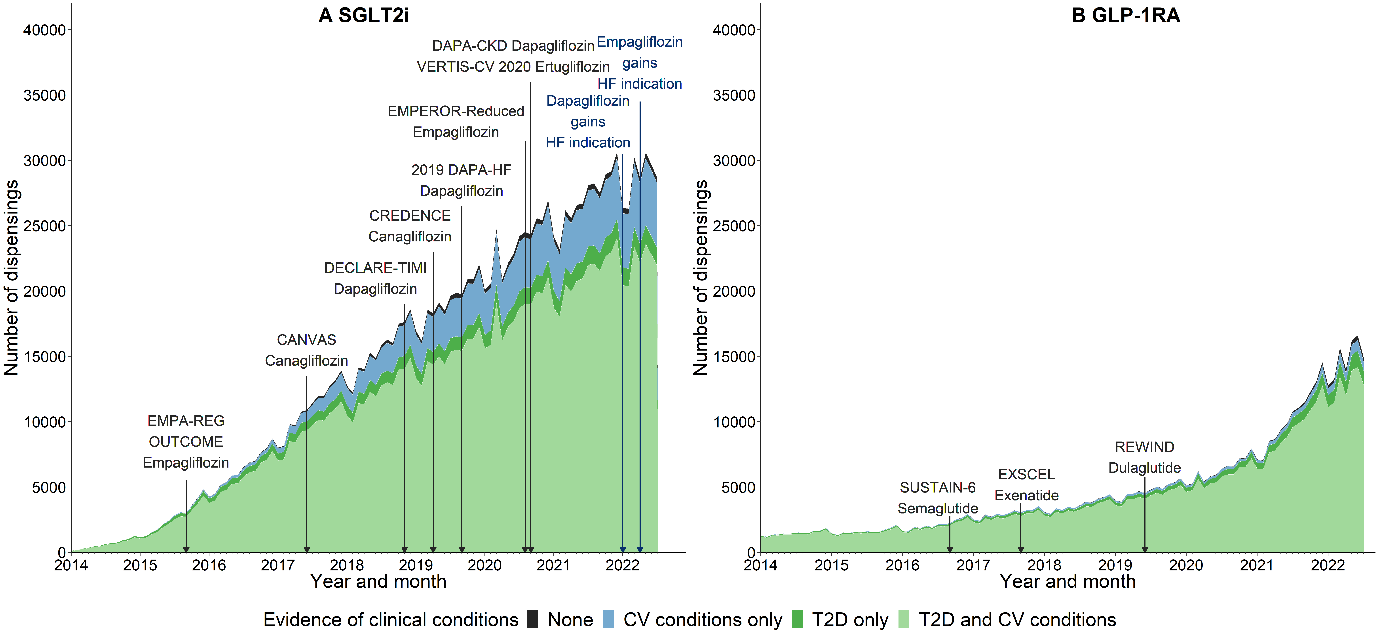
**

SGLT2i, Sodium-glucose co-transporter 2 inhibitors; GLP-1RA, Glucagon-like peptide-1 receptor agonists; CV, Cardiovascular; T2D, Type 2 diabetes.

See Table S1 for the details of each clinical trial.

**Fig. S2** Monthly trends in the number of dispensings by specialty of prescriber

**
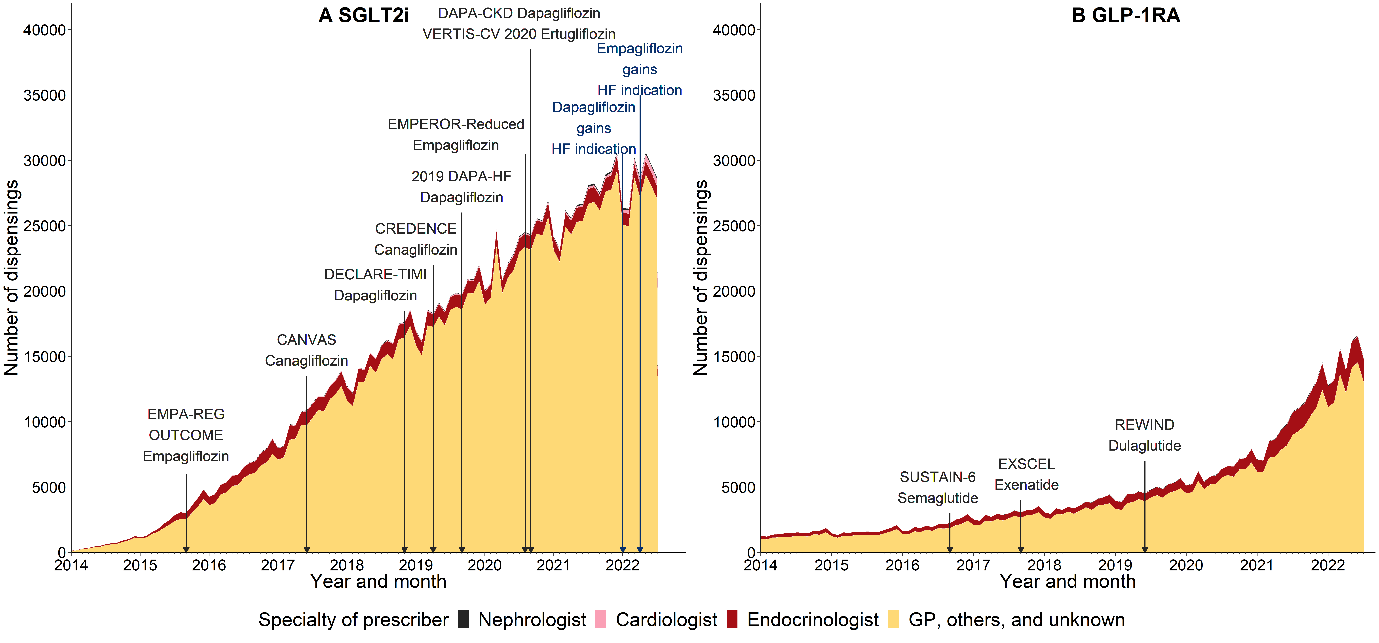
**

SGLT2i, Sodium-glucose co-transporter 2 inhibitors; GLP-1RA, Glucagon-like peptide-1 receptor agonists; GP, General practitioner.

See Table S1 for the details of each clinical trial.

**Table S1** Clinical trials with cardiovascular outcomes for SGLT2i and GLP-1RA during the study period

| **Medicine (Publication reference); Trial name** | **Online publication date** | **Study population and subgroups** | **Cardiovascular outcomes (HR and 95% CI)** |
| --- | --- | --- | --- |
| **SGLT2i** | | | |
| **Empagliflozin^1,2^** | | | |
| EMPA-REG OUTCOME, Cardiovascular Outcome Event Trial in Type 2 Diabetes Mellitus Patients | 17 Sep 2015 | Patients with T2D | Primary composite outcome^*^ (HR=0.86; 95% CI, 0.74-0.99);  Hospitalization for heart failure (HR=0.65; 95% CI, 0.50-0.85) |
|  |  | Patients with T2D and only cerebrovascular disease | Primary composite outcome^*^ (HR=1.15; 95% CI, 0.74-1.78) |
|  |  | Patients with T2D and only coronary artery disease | Primary composite outcome^*^ (HR=0.83; 95% CI, 0.68-1.02) |
|  |  | Patients with T2D and only peripheral artery disease | Primary composite outcome^*^ (HR=0.94; 95% CI, 0.47-1.88) |
|  |  | Patients with T2D and 2 or 3 high CV risk categories | Primary composite outcome^*^ (HR=0.79; 95% CI, 0.61-1.04) |
| EMPEROR-Reduced, Empagliflozin Outcome Trial in Patients With Chronic Heart Failure With Reduced Ejection Fraction | 28 Aug 2020 | Patients with heart failure | Worsening heart failure^§^ or CV death (HR=0.75; 95% CI, 0.65-0.86);  Hospitalization for heart failure (HR=0.69; 95% CI, 0.59-0.81 ) |
|  |  | Patients with heart failure and T2D | Worsening heart failure^§^ or CV death (HR=0.72; 95% CI, 0.60-0.87) |
|  |  | Patients with heart failure without T2D | Worsening heart failure^§^ or CV death (HR=0.78; 95% CI, 0.64-0.97) |
| **Canagliflozin^3,4^** | | | |
| CANVAS, The Canagliflozin Cardiovascular Assessment Study | 12 Jun 2017 | Patients with T2D and high CV risk | Primary composite outcome^*^ (HR=0.86; 95% CI, 0.75-0.97);  Hospitalization for heart failure (HR=0.67; 95% CI, 0.52-0.87) |
|  |  | Patients with T2D and CVD | Primary composite outcome^*^ (HR=0.82; 95% CI, 0.72-0.95) |
|  |  | Patients with T2D without CVD | Primary composite outcome^*^ (HR=0.98; 95% CI, 0.74-1.30) |
|  |  | Patients with T2D and heart failure | Primary composite outcome^*^ (HR=0.80; 95% CI, 0.61-1.05) |
|  |  | Patients with T2D without heart failure | Primary composite outcome^*^ (HR=0.87; 95% CI, 0.76-1.01) |
| CREDENCE, Canagliflozin and Renal Events in Diabetes with Established Nephropathy Clinical Evaluation | 14 Apr 2019 | Patients with T2D and  albuminuric CKD | Primary composite outcome^*^ (HR=0.80; 95% CI, 0.67-0.95);  Hospitalization for heart failure (HR=0.61; 95% CI, 0.47-0.80) |
| **Dapagliflozin^5-7^** | | | |
| DECLARE–TIMI, The Dapagliflozin Effect on Cardiovascular Events–Thrombolysis in Myocardial Infarction | 10 Nov 2018 | Patients with T2D and had or were at risk for atherosclerotic CVD | Primary composite outcome^*^ (HR=0.93; 95% CI, 0.84-1.03);  CV death or hospitalisation for heart failure (HR=0.83; 95% CI, 0.73-0.95);  Hospitalization for heart failure (HR=0.73; 95% CI, 0.61-0.88) |
|  |  | Patients with T2D and atherosclerotic CVD | Primary composite outcome^*^ (HR=0.90; 95% CI, 0.79-1.02);  CV death or hospitalisation for heart failure (HR=0.83; 95% CI, 0.71-0.98) |
|  |  | Patients with T2D and at risk for atherosclerotic CVD | Primary composite outcome^*^ (HR=1.01; 95% CI, 0.86-1.20);  CV death or hospitalisation for heart failure (HR=0.84; 95% CI, 0.67-1.04) |
|  |  | Patients with T2D and heart failure | Primary composite outcome^*^ (HR=1.01; 95% CI, 0.81-1.27);  CV death or hospitalisation for heart failure (HR=0.79; 95% CI, 0.63-0.99) |
|  |  | Patients with T2D without heart failure | Primary composite outcome^*^ (HR=0.92; 95% CI, 0.82-1.02);  CV death or hospitalisation for heart failure (HR=0.84; 95% CI, 0.72-0.99) |
| 2019 DAPA-HF, Dapagliflozin in Patients with Heart Failure and Reduced Ejection Fraction | 19 Sep 2019 | Patients with heart failure | Worsening heart failure^§^ or CV death (HR=0.74; 95% CI, 0.65-0.85);  Hospitalization for heart failure (HR=0.70; 95% CI, 0.59-0.83) |
|  |  | Patients with heart failure and T2D | Worsening heart failure^§^ or CV death (HR=0.75; 95% CI, 0.63-0.90) |
|  |  | Patients with heart failure without T2D | Worsening heart failure^§^ or CV death (HR=0.73; 95% CI, 0.60-0.88) |
| DAPA-CKD trial, Dapagliflozin in Patients with Chronic Kidney Disease | 24 Sep 2020 | Patients with CKD | Death from CV causes^‡^ (HR=0.81; 95% CI 0.58–1.12)  Death for CV outcomes or hospitalization for heart failure (HR=0.71; 95% CI, 0.55-0.92) |
| **Ertugliflozin^8^** | | | |
| VERTIS-CV 2020, Cardiovascular Outcomes with Ertugliflozin in Type 2 Diabetes | 23 Sep 2020 | Patients with T2D and atherosclerotic CVD | Primary composite outcome^*^ (HR=0.97; 95% CI, 0.85-1.11);  Hospitalization for heart failure (HR=0.70; 0.54-0.90) |
| **GLP-1RA** | | | |
| **Semaglutide^9^** | | | |
| SUSTAIN-6, Trial to Evaluate Cardiovascular and Other Long-term Outcomes With Semaglutide in Subjects With Type 2 Diabetes | 16 Sep 2016 | Patients with T2D | Primary composite outcome^*^ (HR=0.74; 95% CI, 0.58-0.95);  Hospitalization for heart failure (HR=1.10; 95% CI, 0.77-1.61) |
|  |  | Patients with T2D and CVD | Primary composite outcome^*^ (HR=0.72; 95% CI, 0.55-0.93) |
|  |  | Patients with T2D and CV risk factors | Primary composite outcome^*^ (HR=1.00; 95% CI, 0.41-2.46) |
|  |  | Patients with T2D and heart failure | Primary composite outcome^*^ (HR=1.03; 95% CI, 0.64-1.66) |
|  |  | Patients with T2D without heart failure | Primary composite outcome^*^ (HR=0.64; 95% CI, 0.48-0.86) |
| **Exenatide^10^** | | | |
| EXSCEL, Exenatide Study of Cardiovascular Event Lowering | 14 Sep 2017 | Patients with T2D | Primary composite outcome^*^ (HR=0.91; 95% CI, 0.83-1.00);  Hospitalization for heart failure (HR=0.94; 95% CI, 0.78-1.13) |
|  |  | Patients with T2D and CV events | Primary composite outcome^*^ (HR=0.90; 95% CI, 0.82-1.00) |
|  |  | Patients with T2D without CV events | Primary composite outcome^*^ (HR=0.99; 95% CI, 0.77-1.28) |
|  |  | Patients with T2D and heart failure | Primary composite outcome^*^ (HR=0.97; 95% CI, 0.81-1.16) |
|  |  | Patients with T2D without heart failure | Primary composite outcome^*^ (HR=0.90; 95% CI, 0.81-1.00) |
| **Dulaglutide^11^** | | | |
| REWIND, Researching Cardiovascular Events with a Weekly Incretin in Diabetes | 10 Jun 2019 | Patients with T2D | Primary composite outcome^*^ (HR=0.88; 95% CI, 0.79-0.99);  Hospitalization for heart failure (HR=0.93; 95% CI, 0.77-1.12) |
|  |  | Patients with T2D and CVD | Primary composite outcome^*^ (HR=0.87; 95% CI, 0.74-1.02) |
|  |  | Patients with T2D without CVD | Primary composite outcome^*^ (HR=0.87; 95% CI, 0.74-1.02) |

SGLT2i, Sodium-glucose co-transporter 2 inhibitors; GLP-1RA, Glucagon-like peptide-1 receptor agonists; HR, Hazard ratio; CI, Confidence interval; T2D, Type 2 diabetes; CVD, Cardiovascular disease; CKD, Chronic kidney disease.

^*^ Primary composite outcome was the first occurrence of death from cardiovascular causes, nonfatal myocardial infarction, or nonfatal stroke.

^§^ Hospitalization or an urgent visit resulting in intravenous therapy for heart failure.

^‡^ Death due to acute myocardial infarction, sudden cardiac death, heart failure, cardiogenic shock, stroke, cardiovascular procedures, or cardiovascular haemorrhage.

**Table S1 References**

1 Zinman B, Wanner C, Lachin JM, et al. Empagliflozin, Cardiovascular Outcomes, and Mortality in Type 2 Diabetes. N Engl J Med. 2015;373(22):2117-2128.

2 Packer M, Anker SD, Butler J, et al. Cardiovascular and Renal Outcomes with Empagliflozin in Heart Failure. N Engl J Med. 2020;383(15):1413-1424.

3 Neal B, Perkovic V, Mahaffey KW, et al. Canagliflozin and Cardiovascular and Renal Events in Type 2 Diabetes. N Engl J Med. 2017;377(7):644-657.

4 Perkovic V, Jardine MJ, Neal B, et al. Canagliflozin and Renal Outcomes in Type 2 Diabetes and Nephropathy. N Engl J Med. 2019;380(24):2295-2306.

5 Wiviott SD, Raz I, Bonaca MP, et al. Dapagliflozin and Cardiovascular Outcomes in Type 2 Diabetes. N Engl J Med. 2019;380(4):347-357.

6 McMurray JJV, Solomon SD, Inzucchi SE, et al. Dapagliflozin in Patients with Heart Failure and Reduced Ejection Fraction. N Engl J Med. 2019;381(21):1995-2008.

7 Heerspink HJL, Stefánsson BV, Correa-Rotter R, et al. Dapagliflozin in Patients with Chronic Kidney Disease. N Engl J Med. 2020;383(15):1436-1446.

8 Cannon CP, Pratley R, Dagogo-Jack S, et al. Cardiovascular Outcomes with Ertugliflozin in Type 2 Diabetes. N Engl J Med. 2020;383(15):1425-1435.

9 Marso SP, Bain SC, Consoli A, et al. Semaglutide and Cardiovascular Outcomes in Patients with Type 2 Diabetes. N Engl J Med. 2016;375(19):1834-1844.

10 Holman RR, Bethel MA, Mentz RJ, et al. Effects of Once-Weekly Exenatide on Cardiovascular Outcomes in Type 2 Diabetes. N Engl J Med. 2017;377(13):1228-1239.

11 Gerstein HC, Colhoun HM, Dagenais GR, et al. Dulaglutide and cardiovascular outcomes in type 2 diabetes (REWIND): a double-blind, randomised placebo-controlled trial. The Lancet. 2019;394(10193):121-130.

**Table S2** Codes for identifying medicines for SGLT2i and GLP-1RA, by therapeutic indication

| **Medicine (WHO ATC code)** | **PBS item code** | **Time of the first listing** | **Estimated shortage period^†^** |
| --- | --- | --- | --- |
| **SGLT2i** |  |  |  |
| Canagliflozin (A10BX11) |  |  |  |
| Type 2 diabetes^1,2^ | 02987F, 02873F | Dec 2013 – Aug 2015^*^ |  |
| Dapagliflozin (A10BK01) |  |  |  |
| Type 2 diabetes^3^ | 10011X, 11291G | Dec 2013 |  |
| Heart failure^4^ | 12823X | Jan 2022 |  |
| Chronic kidney disease^5^ | 13106T | Sep 2022 |  |
| Dapagliflozin + metformin (A10BD15) |  |  | Nov 2017 |
| Type 2 diabetes^3^ | 10510E, 10515K, 10516L, 1270E,11300R, 11313K | Oct 2015 |  |
| Dapagliflozin + saxagliptin (A10BD21) |  |  |  |
| Type 2 diabetes^3^ | 11286B, 11305B | Apr 2018 |  |
| Empagliflozin (A10BK03) |  |  |  |
| Type 2 diabetes^6^ | 10202Y, 10206E, 11281R, 11314L | Jan 2015 |  |
| Heart failure^7^ | 12918X | Apr 2022 |  |
| Empagliflozin + linagliptin (A10BD19) |  |  |  |
| Type 2 diabetes^6^ | 11269D, 11298P, 11303X, 11310G | Apr 2018 |  |
| Empagliflozin + metformin (A10BD20) |  |  | Aug-Oct 2016, Mar-Apr 2020, Jan-Feb 2022 |
| Type 2 diabetes^6^ | 10626G, 10627H, 10633P, 10639Y, 10640B, 10649L, 10650M, 10677Y | Mar 2016 |  |
| Ertugliflozin (A10BK04) |  |  |  |
| Type 2 diabetes^8^ | 11570Y, 11571B, 11577H, 11585R | Dec 2018 |  |
| Ertugliflozin + metformin (A10BD23) |  |  | Jun-Aug 2022 |
| Type 2 diabetes^8^ | 11562M, 11563N, 11564P, 11568W, 11569X, 11575F, 11581M, 11584Q | Dec 2018 |  |
| Ertugliflozin + sitagliptin (A10BD24) |  |  | Sep 2021 |
| Type 2 diabetes^8^ | 11561L, 11578J, 11579K, 11583P | Dec 2018 |  |
| **GLP-1RA** |  |  |  |
| Exenatide (A10BJ01) |  |  | Jul 2014, May-Jun 2016, Aug 2021 |
| Type 2 diabetes^9^ | 03423E, 03424F, 10888C | Aug 2010 |  |
| Dulaglutide (A10BJ05) |  |  | Jun 2022-Mar 2023 |
| Type 2 diabetes^10^ | 11364D | Jun 2018 |  |
| Semaglutide (A10BJ06) |  |  | Apr 2022-Mar 2023 |
| Type 2 diabetes^11^ | 12075M, 12080T | Jul 2020 |  |

SGLT2i, Sodium-glucose co-transporter 2 inhibitors; GLP-1RA, Glucagon-like peptide-1 receptor agonists; WHO, World Health Organisation; ATC, Anatomic Therapeutic Chemical; PBS, Pharmaceutical Benefits Scheme.

^*^ Canagliflozin was delisted from the PBS in August 2015.

^†^ Information available from the Medicine shortage reports database of the Therapeutical Goods Administration^12^.

**Table S2 References**

1 NPS MedicineWise. Canagliflozin for type 2 diabetes. Aust Prescr. 2013;37:28-35.

2 Australian Government Department of Health. Schedule of Pharmaceutical Benefits \u2013 Effective 1 March 2015. <https://www.pbs.gov.au/publication/schedule/2015/03/2015-03-01-general-schedule-soc.pdf>. Published 2015. Accessed 7 June 2023.

3 NPS MedicineWise. Dapagliflozin for type 2 diabetes. Aust Prescr. 2013;36:174-179.

4 NPS MedicineWise. Dapagliflozin (Forxiga) for heart failure with reduced ejection fraction (LVEF ≤ 40%). NPS RADAR. <https://www.nps.org.au/radar/articles/dapagliflozin-forxiga-for-heart-failure-with-reduced-ejection-fraction-lvef-40#r22>. Published 2021. Accessed 7 June 2023.

5 NPS MedicineWise. Dapagliflozin for chronic kidney disease. NPS RADAR. <https://www.nps.org.au/radar/articles/dapagliflozin-for-chronic-kidney-disease>. Published 2022. Accessed 7 June 2023.

6 NPS MedicineWise. Empagliflozin for type 2 diabetes. Aust Prescr. 2015;38:64-66.

7 NPS MedicineWise. Empagliflozin (Jardiance) for heart failure with reduced ejection fraction (LVEF ≤ 40%). NPS RADAR. <https://www.nps.org.au/radar/articles/empagliflozin-jardiance-for-heart-failure-with-reduced-ejection-fraction-lvef-40>. Published 2022. Accessed 7 June 2023.

8 NPS MedicineWise. Ertugliflozin for type 2 diabetes. Aust Prescr. 2019;42:70-72.

9 NPS MedicineWise. Exenatide (Byetta) for type 2 diabetes mellitus. NPS RADAR. <https://www.nps.org.au/radar/articles/exenatide-byetta-for-type-2-diabetes-mellitus>. Published 2010. Accessed 7 June 2023.

10 NPS MedicineWise. Dulaglutide for type 2 diabetes. Aust Prescr 2018;2018(41):166-168.

11 NPS MedicineWise. Semaglutide for type 2 diabetes. Aust Prescr. 2020;43:137-139.

12 Australian Government Department of Health and Aged Care, Therapeutical Goods Administration. Medicine Shortage reports database. <https://apps.tga.gov.au/Prod/msi/search?shortagetype=All>. Accessed 7 June 2023.

**Table S3** Anatomical Therapeutic Chemical (ATC) codes for identifying history of medicine use and morbidities in the year prior including the date of initiation

| Medicine use and morbidities (definition) | ATC code |
| --- | --- |
| Type 2 diabetes (Use of blood glucose-lowering agents other than sodium-glucose co-transporter 2 inhibitors or glucagon-like peptide-1 receptor agonists, but those who used insulin only were considered as type 1 diabetes) | |
| Insulin | A10AA-A10AE |
| Metformin | A10BA02, A10BD02, A10BD03, A10BD07, A10BD08, A10BD10, A10BD11, A10BD13 |
| Sulfonylureas | A10BB, A10BD02 |
| Dipeptidyl peptidase-4 inhibitors | A10BH, A10BD07, A10BD08, A10BD10, A10BD11, A10BD13, A10BD19, A10BD21, A10BD24 |
| Others (Thiazolidinediones, acarbose) | A10BG, A10BD03, A10BF |
| Cardiovascular conditions (Use of cardiovascular medicines) | |
| Antihypertensives | C03A, C03BA04, C03BA11, C08, C09, C10BX |
| Lipid-lowering agents | C10 |
| Anticoagulants | B01A |
| Antiplatelets | B01AC |
| Beta-blockers | C07 |
| Loop diuretics | C03C |
| Vasodilators | C01D |
| Potassium-sparing diuretics | C03D, C03E |
| Antiarrhythmics | C01B |
| Glycosides | C01AA |
| Other cardiovascular groups | C01EB17, C02AB, C02AC, C02CA, C03DB, C02DC |
| Anticoagulants^*^ (Use of anticoagulants) | |
| Anticoagulants | B01A |
| Antiplatelets^*^ (Use of antiplatelets) | |
| Antiplatelets | B01AC |
| Arrhythmia^*^ (Use of medicines treating arrhythmia) | |
| Digoxin | C01AA05 |
| Antiarrhythmics | C01B |
| Sotalol | C07AA07 |
| Congestive heart failure^*^ (Use of medicines treating congestive heart failure) | |
| Aldosterone antagonists | C03DA |
| Beta-blockers | C07AB02, C07AB07, C07AG02, C07AB12 |
| Diuretics AND Renin-angiotensin-aldosterone system inhibitors | (C03C) AND (C09A OR C09C) |
| Hyperlipidaemia^*^ (Use of lipid-lowering agents) | |
| Lipid-lowering agents | C10, A10BH03 |
| Hypertension^*^ (Use of medicines treating hypertension) | |
| Antihypertensives | C03AA01-C03BA11, C03DB01, C03DB99, C03EA01, C09BA, C09DA |
| Antiadrenergic agents | C02A |
| Hydrazinophthalazine derivatives | C02DB |
| Loop diuretics OR angiotensin-converting enzyme inhibitors | (C03C) OR (C09A) |
| Ischaemic heart disease: angina^*^ (Use of medicines treating ischaemic heart disease: angina) | |
| Vasodilators used in cardiac diseases | C01D, C08EX02 |
| Ischaemic heart disease: hypertension^*^ (Use of medicines treating ischaemic heart disease: hypertension) | |
| Beta-blockers | C07 |
| Calcium channel blockers | C08 |
| Angiotensin receptor blockers AND calcium channel blockers | C09D, C09DX01, C09DX03 |
| Angiotensin-converting enzyme inhibitors AND calcium channel blockers | C09BB02-C09BB10, C10BX03 |
| Renal disease^*^ (Use of medicines treating renal disease) | |
| Antianemics other than iron, vitamin B12 and folic acid | B03XA |
| Vitamin D and analogues | A11CC |
| Serotonin receptor antagonists | V03AE |

^*^ Reproduced from Pratt NL, Kerr M, Barratt JD, Kemp-Casey A, Kalisch Ellett LM, Ramsay E, et al. The validity of the Rx-Risk Comorbidity Index using medicines mapped to the Anatomical Therapeutic Chemical (ATC) Classification System. BMJ Open. 2018;8(4):e021122.
